# Laparoscopic liver cyst fenestration with real-time indocyanine green fluorescence-guided surgery: a case report

**DOI:** 10.1093/jscr/rjab196

**Published:** 2021-05-17

**Authors:** Norikazu Une, Atsushi Fujio, Hiroaki Mitsugashira, Norifumi Kanai, Yoshikatsu Saitoh, Mineto Ohta, Kengo Sasaki, Koji Miyazawa, Toshiaki Kashiwadate, Wataru Nakanishi, Kazuaki Tokodai, Shigehito Miyagi, Michiaki Unno, Takashi Kamei

**Affiliations:** Department of Surgery, Tohoku University Graduate School of Medicine, Sendai, Miyagi, Japan; Department of Surgery, Tohoku University Graduate School of Medicine, Sendai, Miyagi, Japan; Department of Surgery, Tohoku University Graduate School of Medicine, Sendai, Miyagi, Japan; Department of Surgery, Tohoku University Graduate School of Medicine, Sendai, Miyagi, Japan; Department of Surgery, Tohoku University Graduate School of Medicine, Sendai, Miyagi, Japan; Department of Surgery, Tohoku University Graduate School of Medicine, Sendai, Miyagi, Japan; Department of Surgery, Tohoku University Graduate School of Medicine, Sendai, Miyagi, Japan; Department of Surgery, Tohoku University Graduate School of Medicine, Sendai, Miyagi, Japan; Department of Surgery, Tohoku University Graduate School of Medicine, Sendai, Miyagi, Japan; Department of Surgery, Tohoku University Graduate School of Medicine, Sendai, Miyagi, Japan; Department of Surgery, Tohoku University Graduate School of Medicine, Sendai, Miyagi, Japan; Department of Surgery, Tohoku University Graduate School of Medicine, Sendai, Miyagi, Japan; Department of Surgery, Tohoku University Graduate School of Medicine, Sendai, Miyagi, Japan; Department of Surgery, Tohoku University Graduate School of Medicine, Sendai, Miyagi, Japan

**Keywords:** Hepatobiliary surgery

## Abstract

Laparoscopic fenestration (LF) has recently been considered a standard procedure for nonparasitic symptomatic liver cysts. Here, we report a case of LF that was safely performed using real-time indocyanine green (ICG) fluorescence-guided surgery. A 74-year-old woman presented with right upper abdominal pain and poor dietary intake. The patient was diagnosed with symptomatic liver cysts and underwent LF. One hour before surgery, ICG (2.5 mg) was intravenously administered to the patient. ICG fluorescence imaging clearly showed the biliary ducts and distinguished the cysts from the liver parenchyma. We could resect only the cyst walls as wide as possible under the guidance of both white light and fluorescence imaging. There were no signs of postoperative symptom recurrence. Detection of ICG fluorescence in the liver parenchyma is as important as ICG cholangiography for fenestration. Laparoscopic liver cyst fenestration with real-time ICG fluorescence-guided surgery is safe and can be used as a standard procedure.

## INTRODUCTION

Liver cysts are a relatively common disease, and laparoscopic fenestration (LF) has recently been considered a standard procedure for nonparasitic symptomatic liver cysts [1, 2]. The principle of LF is to resect the cyst wall as wide as possible at the cyst–liver boundary to prevent recurrence. However, excessive resection of the cyst wall may induce severe complications, such as bleeding and bile leakage [3]. A recent study has reported it is feasible and safe to use indocyanine green (ICG) for evaluating tissue blood flow and identifying biliary tracts [4]. In hepatobiliary surgery, intraoperative fluorescent cholangiography using ICG can avoid bile duct injury during laparoscopic cholecystectomy [5]. In addition, intraoperative ICG fluorescence imaging can detect liver tumours due to the difference in the hepatobiliary excretion capacity of ICG between the liver tumour and liver parenchyma [6]. However, there are few reports of LF using ICG fluorescence imaging.

Here, we report a case in which laparoscopic liver cyst fenestration was safely performed using real-time intraoperative ICG fluorescence imaging.

## CASE REPORT

**Figure 1:**
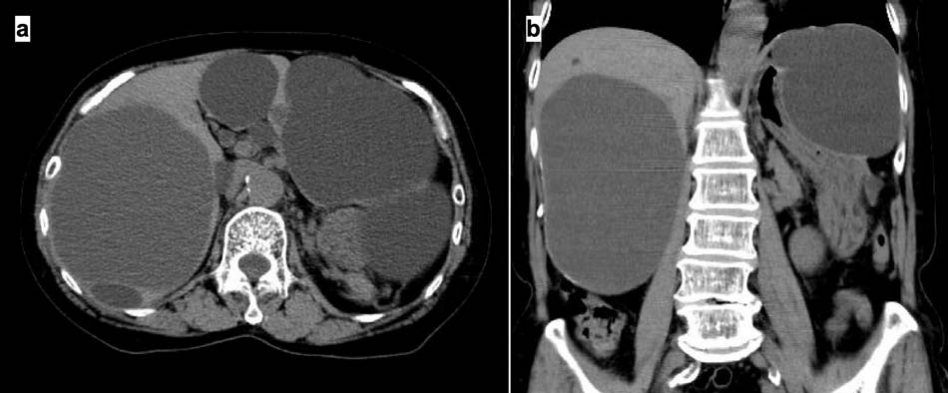
Preoperative CT imaging demonstrating multiple large liver cysts in the bilateral liver lobe and compression of the stomach due to a large cyst (**a**: axial section, **b**: coronal section).

**Figure 2:**
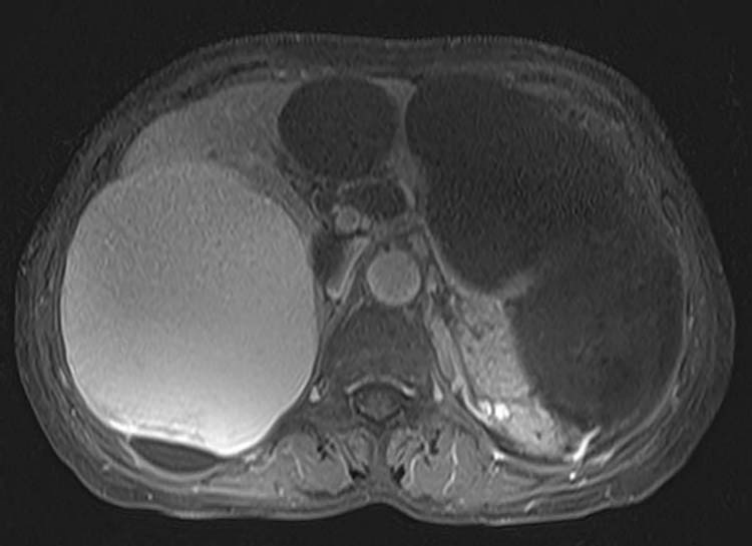
Preoperative T1-weighted magnetic resonance imaging showing different findings in the right and left lobe cysts.

**Figure 3:**
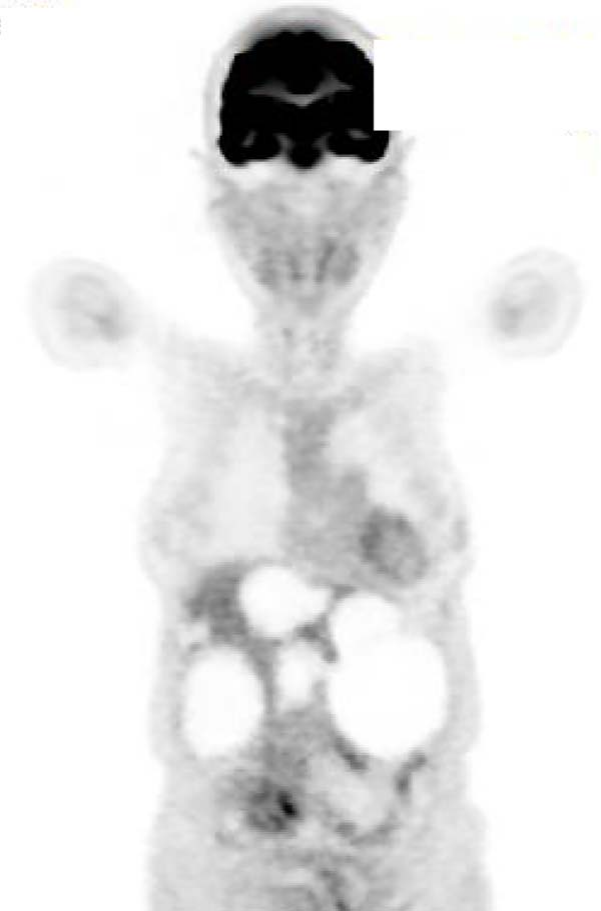
Positron emission tomography-CT showing no increase in ^18^F-fluorodeoxyglucose metabolism in the liver cysts.

**Figure 4:**
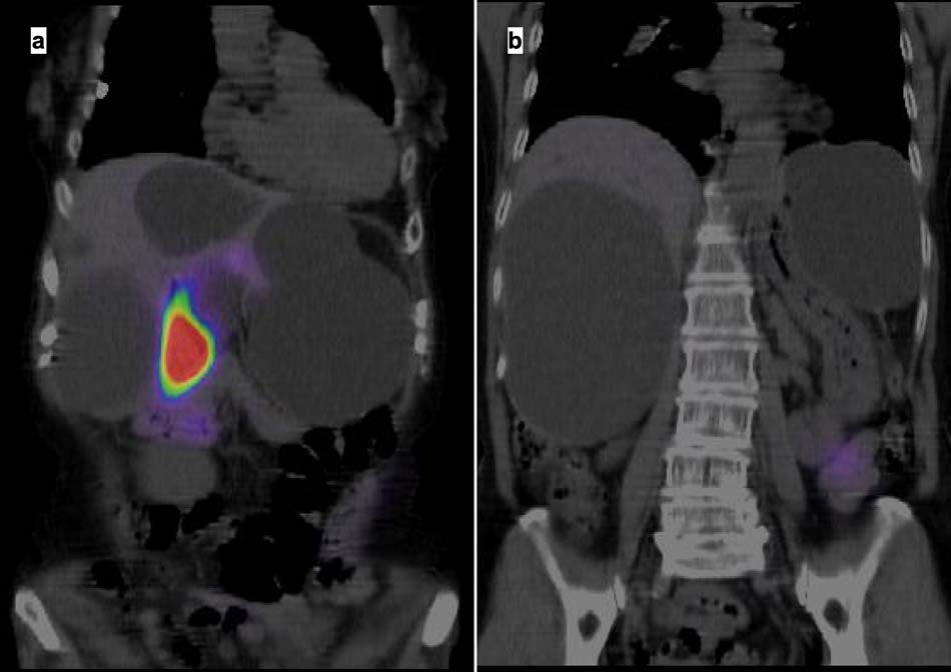
Hepatobiliary scintigraphy with CT highlighting only the gallbladder, with no biliary communication with the liver cysts.

A 74-year-old woman presented with right upper abdominal pain and poor dietary intake. She had no notable medical history or history of abdominal trauma. Abdominal computed tomography (CT) showed multiple liver cysts, with the size of the largest cyst in the right lobe measuring 155 × 113 × 106 mm (Fig. 1a). In addition, a large cyst measuring 135 mm in the left lateral lobe strongly compressed the stomach (Fig. 1b). T1-weighted magnetic resonance imaging revealed different findings in the right and left lobe cysts, and there were no enhanced structures in the liver cysts (Fig. 2). Positron emission tomography-CT did not show increased ^18^F-fluorodeoxyglucose metabolism in any of the liver cysts (Fig. 3). Moreover, hepatobiliary scintigraphy with CT showed no biliary communication with the liver cysts (Fig. 4). The patient was diagnosed with symptomatic and multiple liver cysts with no biliary communication or malignancy. Therefore, LF was performed.

**Figure 5:**
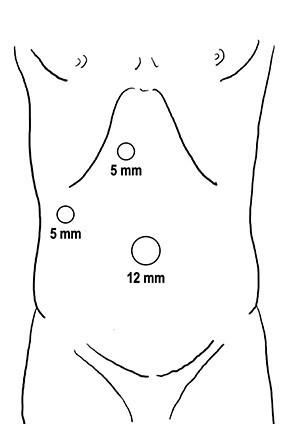
Trocar placement.

**Figure 6:**
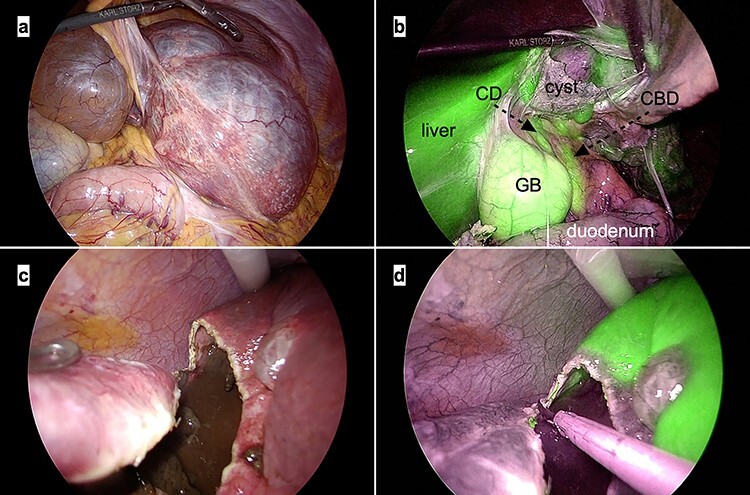
Intraoperative laparoscopic images. (**a**) The large cysts in the left lateral lobe can be seen compressing the stomach; (**b**) Indocyanine green fluorescence is observed in the liver parenchyma and biliary tracts but not in the cyst wall. *Abbreviations:* CBD, common bile duct; CD, cystic duct; GB, gallbladder; (**c** and **d**) The cyst wall is cut precisely at the cyst–liver boundary under the guidance of white light (c) and ICG fluorescence imaging (d).

One hour before surgery, the patient (body weight: 45.9 kg) was intravenously administered ICG (2.5 mg). A 12-mm trocar was placed at the umbilicus for scope entry, and two 5-mm ports were placed at the epigastric and right upper areas to manipulate the instruments (Fig. 5). A laparoscopic imaging system (Visera Elite II; Olympus, Tokyo, Japan) was used to detect ICG fluorescence. Large cysts were observed in the bilateral lobe, and large cysts in the left lateral lobe compressed the stomach (Fig. 6a). ICG fluorescence was detected in the liver parenchyma and the biliary tract, but not in the cyst wall (Fig. 6b). The left and right lobe cysts were punctured to drain fluid. Biochemical examination showed no elevation of bilirubin levels in the cystic fluid from either the left or right lobe cysts. ICG fluorescence imaging clearly distinguished the cysts from the liver parenchyma, and we could resect only the cyst wall as wide as possible under the guidance of white light and fluorescence imaging (Fig. 6c and d). Intracystic bile ducts and bile leaks in the cysts were not detected during surgery. The large cysts were completely fenestrated, and the small cysts were conserved. The total surgical time was 73 min. The total cystic fluid volume was 1900 ml, and there was no blood loss during surgery. All cysts were histopathologically diagnosed as benign liver cysts lined with cuboidal epithelium.

The patient had no complications and recovered quickly; she was discharged on postoperative Day 6. The patient’s condition was good, and there was no recurrence of the preoperative symptoms after discharge.

## DISCUSSION

Liver cysts are relatively common, and their prevalence has increased from 2.5 to 5% in the 1990s to 18% in recent years [7, 8]. Although most hepatic cysts are asymptomatic, large cysts may induce symptoms such as mechanical discomfort, pain, nausea and vomiting. Symptomatic liver cysts may require surgical intervention, including simple drainage, sclerotherapy, open or LF and liver resection. There are several treatments for liver cysts; however, cyst aspiration with and without sclerotherapy has a high risk of cyst recurrence; therefore, it is generally not recommended as a first-line treatment [9].

Recently, minimally invasive surgery and intraoperative real-time imaging using ICG have been widely used in hepatobiliary surgery. LF is considered a standard procedure for the treatment of symptomatic nonparasitic hepatic cysts [1, 2]. In a systematic review, compared with the open surgical approach, the laparoscopic approach significantly reduced the operation time and length of hospital stay. Furthermore, the review reported a lower recurrence rate with the laparoscopic approach than with the open surgical approach [10].

Several studies have shown that intraoperative fluorescent cholangiography using ICG is a safe and useful method for the prevention of bile duct injury in LF [11–14]. Although these studies have mainly focused on cholangiography using ICG fluorescence, we believe that detection of ICG fluorescence in the liver parenchyma is as important as ICG cholangiography for fenestration. For example, in patients with large liver cysts, we sometimes encounter significantly thin liver parenchyma, and it may be difficult to distinguish the boundary between the cysts and liver parenchyma. In addition, it is often difficult to detect intracystic bile ducts on preoperative imaging. We could clearly distinguish the cyst wall from the liver parenchyma and biliary tracts during surgery using white light and fluorescence imaging, which helped us to perform wide and safe cyst fenestration. We believe that LF with real-time ICG fluorescence-guided surgery reduces bleeding and bile leakage. Moreover, administration of only 2.5 mg of ICG (~0.05 mg) 1-h before surgery was sufficient to visualise the vivid fluorescence in the biliary tracts and liver parenchyma. However, it is expected that ICG fluorescence levels in the liver parenchyma and biliary tracts may vary depending on the ICG dose, timing of ICG administration, differences in laparoscopic fluorescence imaging systems, and liver function. Hence, further investigations on ICG imaging in hepatobiliary surgery are needed.

In conclusion, laparoscopic liver cyst fenestration with real-time ICG fluorescence-guided surgery is safe and can be used as a standard procedure.

## CONFLICTS OF INTEREST STATEMENT

The authors declare that they have no conflicts of interests.

## FUNDING

The authors declare that they have not received any funding for this study.
